# Investigation on the Loss of Taste and Smell and Consequent Psychological Effects: A Cross-Sectional Study on Healthcare Workers Who Contracted the COVID-19 Infection

**DOI:** 10.3389/fpubh.2021.666442

**Published:** 2021-05-28

**Authors:** Luisa Dudine, Claudia Canaletti, Fabiola Giudici, Alberta Lunardelli, Giulia Abram, Ingrid Santini, Vera Baroni, Marta Paris, Valentina Pesavento, Paolo Manganotti, Federico Ronchese, Barbara Gregoretti, Corrado Negro

**Affiliations:** ^1^Clinic of Psychology, Department of Hospital Care, University Hospital and Health Services, Trieste, Italy; ^2^Unit of Biostatistics, Department of Cardiac, Thoracic, Vascular Sciences and Public Health, Epidemiology and Public Health, University of Padua, Padua, Italy; ^3^Rehabilitation Division, Department of Integrated Neuroscience and Occupational Medicine, University Hospital and Health Services, Trieste, Italy; ^4^Clinical Unit of Neurology, Department of Medicine, Surgery and Health Sciences, University Hospital and Health Services of Trieste, University of Trieste, Trieste, Italy; ^5^Clinical Unit of Occupational Medicine, Department of Integrated Neuroscience and Occupational Medicine, University Hospital and Health Services, Trieste, Italy; ^6^Medical Directorate, University Hospital and Health Services, Trieste, Italy; ^7^Clinical Unit of Occupational Medicine, University of Trieste, Trieste, Italy

**Keywords:** COVID-19, taste disorder, smell loss, psychological distress, healthcare workers

## Abstract

The aim of this study was to investigate the correlation between psychological distress and taste and sense of smell dysfunctions on healthcare workers (HCW) who contracted the COVID-19 infection in the midst of the disease outbreak. Reports of sudden loss of taste and smell which persist even after recovery from COVID-19 infection are increasingly recognized as critical symptoms for COVID-19 infections. Therefore, we conducted a cross-sectional study on COVID-19 HCW (*N* = 104) who adhered to respond to a phone semistructured interview addressing the virus symptoms and associated psychological distress. Data were collected from June to September 2020. Findings confirm the association between experienced taste/olfactory loss and emotional distress and suggest that dysfunctions of taste and smell correlate positively with anxiety and depression. Furthermore, their psychological impact tends to persist even after the recovery from the disease, suggesting the need for appropriate psychological interventions to prevent people from developing more serious or long-lasting psychological disorders and, as far as HCW, to reduce the risk of work-related distress.

## Introduction

In December 2019, an outbreak of pneumonia with unknown origin began in China's Hubei Province raising global health concerns due to the ease of transmission. After numerous studies conducted around the world, a novel severe acute respiratory syndrome coronavirus 2 (SARS-CoV-2) was identified and was called “coronavirus-19” (COVID-19). For the National Institutes of Health (1), several symptoms characterized this infection, ranging from asymptomatic/mild symptoms to severe illness and death. In particular, COVID-19 symptomatology included cough, fever, and shortness of breath, as well as weakness, malaise, respiratory distress, muscle pain, sore throat, loss of taste, and/or smell (2–8).

In countless studies (9–14), smell dysfunction has been demonstrated as one of the first among other neurologic manifestations of both hospitalized and mild COVID-19 patients (15).

World's pandemics are usually associated with adverse mental health consequences as reported in different studies (16–22). According to Rajkumar (23), first evidence suggests that symptoms of anxiety and depression and self-reported distress are common psychological reactions to the COVID-19 pandemic and may be associated with disturbed sleep.

However, studies on determinants of psychological distress have rarely focused on the clinical manifestation of the SARS-COV-2.

During the first wave of COVID-19 pandemic, a psychology crisis unit (coworked with the Occupational Medicine Service) kept in touch by phone calls with HCW infected with COVID-19 to prevent psychological distress, and observed an association between psychological distress and olfactory/taste dysfunctions.

Therefore, our primary aim was to determine the psychological impact of COVID-19 symptomatology and, more specifically, whether taste/olfactory dysfunctions were associated with more emotional disorders as compared with subjects not experiencing these sense symptomatologies. Indeed, it is well-known that taste and olfactory sensory dysfunctions/loss represent an early manifestation of infection (7, 10, 11, 24) that can have a negative impact on emotional well-being and quality of life (25) people with anosmia often refer feelings of loneliness, fear, and depression as well-difficulties concerning social and sexual relationship, concerns about personal hygiene. Taste disturbance or loss can also cause subjective discomfort and have a negative impact on nutrition, indeed depression subsequent to gustatory dysfunction has been described (26). In addition, in one study, Weiffenabch and Bartoshuk (27) reported how dysfunction of taste and smell can amplify psychological distress. Further analyses concerned the prevalence of smell and taste dysfunctions, how these symptoms were distributed within our sample, and at what stage of the disease they tended to emerge. Because the mental status of health professionals directly affected by the COVID-19 epidemic to our knowledge has been under-addressed, we focus on a sample of HCW who contracted the infection during the first wave of COVID-19. The purpose of the study will also investigate the distress and psychological needs and serve to better target and plan support services for healthcare professionals working in COVID-19 centers, especially focusing on work-related distress. The HCW normally represent an important sector, actually overexposed to COVID-19 consequences, that needs to be further explored and protected. This study represents a started point for future researches.

## Materials and Methods

A no-profit study representing a cross-sectional investigation was conducted from June to September 2020 according to the local Ethical Committee (N.051/2020). HCW of the Trieste area in Northern Italy who contracted the COVID-19 infection in the midst of the disease outbreak were interviewed by phone by psychologists of the same healthcare service. The presence of infection was determined by the positive result of molecular swabs as established by the World Health Organization (28).

### Participants

One hundred twenty-three HCW were involved in the study by occupational doctors from an Italian local network of hospitals. However, the final sample of subjects who adhered to participate to the study was composed by a total of 104 subjects (women *N* = 71 and men *N* = 33) between the ages of 23 and 65 years (mean = 43) who completed the interview and the questionnaires. At the time of the interview, participants were COVID-19 positive, COVID-19 negative with symptoms, and COVID-19 negative without symptoms (see Table 1). The inclusion criteria were age >18–<70; contraction of COVID-19 infection; good understanding of the Italian language; and those who have expressed their favorable consent to participate in the study. The exclusion criterion was inability to cooperate due to high distress at the time of the interview evaluated by the psychologist.

**Table 1:** Participants demographics and clinical characteristics.

	**Total cohort (*n* = 104)**			
**Demographics**				
**Gender**				
Male	33 (31.7%)			
Female	71 (68.3%)			
Age (years)				
Mean (SD)	42 (11)			
Median (min-max)	44 (23–65)			
Patient's status at interview				
COVID-19 positive	5 (4.8%)			
COVID-19 negative with symptoms	38 (36.5%)			
COVID-19 negative without symptoms	61 (58.7%)			
	**All cohort (*****n*** **=** **104)**	**Female (*****n*** **=** **71)**	**Male (*****n*** **=** **33)**	***P*****-value**
**COVID-19 symptoms**				
Fatigue	85 (83.3%)	60 (85.7%)	25 (78.1%)	0.34
Olfactory dysfunctions	84 (81.6%)	62 (88.6%)	22 (66.7%)	0.007
Taste dysfunctions	79 (76.7%)	59 (84.3%)	20 (60.6%)	0.008
General Illness	76 (73.8%)	53 (75.7%)	23 (69.7%)	0.52
Muscle aches	66 (64.1%)	43 (61.4%)	23 (69.7%)	0.41
Nasal Congestion	64 (62.1%)	47 (67.1%)	17 (51.5%)	0.13
Fever	62 (60.2%)	42 (60.0%)	20 (60.6%)	0.95
Headache	52 (50.5%)	37 (52.9%)	15 (45.5%)	0.48
Dry cough	48 (46.6%)	33 (47.1%)	15 (45.5%)	0.87
Sore throat	36 (35.0%)	29 (41.4%)	7 (21.2%)	0.05
Diarrhea	28 (27.2%)	19 (27.1%)	9 (27.3%)	0.99
Fainting	16 (15.5%)	11 (15.7%)	5 (15.2%)	0.94
Numbness	10 (9.7%)	6 (8.6%)	4 (12.2%)	0.57
Difficulty closing eyes	2 (1.9%)	1 (1.4%)	1 (1.3%)	0.54

### Instruments and Procedure

Subjects were reached by phone and administered a semistructured interview which involved questions concerning clinical symptoms of COVID-19 including taste and smell problems, the distress thermometer (29), and the Hospital Anxiety and Depression Scale (HADS) (30, 31). The distress thermometer (DT) is a simple self-reported tool that screens for symptoms of distress and has been found valid in measuring psychological distress in different countries, cultures, and pathological populations as confirmed by different studies (32–34). It measures the stress variable on a 0- (no distress) to 10-rating (extreme distress) scale. The DT can be considered a continuous variable, with an established cutoff of 5 indicating experience of significant distress and need for attention or can be employed to classify people with low (0–3), moderate (4–5), and high levels (>6) of stress, as reported by Grassi et al. (35).

The HADS questionnaire is a 14-item scale designed to detect emotional disturbances in non-psychiatric patients (31) in hospital settings by screening for the two most frequent disorders: anxiety and depression. Each item is rated on a 4-point scale, ranging from 0 to 3, with 3 indicating higher symptom frequency. Two separate subscales identifying anxiety and depression symptoms can be obtained as well as a total score of psychological state. Total scores for each subscale range from 0 to 21, categorized as: normal (0–7), borderline (8–10), and present disorder (≥11).

In the present study, the DT was proposed to participants considering two different periods of time: (1) COVID-DT which refers to the stress perceived during the early illness and (2) Current-DT which indicates the level of stress perceived at the time of the interview about COVID-19. The HADS score refers only to the time of the interview.

### Data Analysis

Taking into account the primary objective of the study, namely the association between olfactory and gustatory dysfunctions and a state of distress, the sample size available (*n* = 104) can be considered sufficient to determine a difference of 0.62 (medium Cohen's *d* effect size) in the outcome evaluation scale (DT) assuming a power of 80% (1-β) and a level of significance of 5% (α) (two-tailed student *t* test).

Categorical variables were expressed by frequencies and percentage, while continuous variables were expressed by mean [standard deviation (SD)] or median [range (min-max)], as appropriate according to data distribution verified through Shapiro Wilk normality test. Student's *t* test or Mann-Whitney test were used to compare continuous variables (age, DT, HADS scores) with respect to groups of interests. Independent Chi-square test or Fischer exact test (when appropriate) were performed to evaluate and investigate associations among categorical variables. Paired Wilcoxon test compared DT values perceived by the patients during COVID-19 infection with current distress. Spearman linear correlation coefficients were calculated in order to verify correlation among different psychological scores. Multivariate linear regression models were performed to identify risk factors for psychological disorders and results reported as Beta coefficient regression with 95% confidence interval. All the statistical tests were two sided, and statistical significance was defined as *p*-value < 0.05. Analyses were performed using statistical software R, v. 4.0.3, 2020.

## Results

### COVID-19 Symptoms

Subjects' response analysis on COVID-19 symptoms revealed that only one person was asymptomatic and three workers required hospitalization, while the majority of the sample experienced mild to moderate symptomatology with an average of 7 symptoms (60.2%). The most frequent symptoms were fatigue (83%), olfactory dysfunctions (82%), and taste dysfunctions (77%) Women reported dysfunctions significantly greater than men in smell (89 vs. 67%, *p* = 0.007), taste (84 vs. 61%, *p* = 0.008), and sore throat (41 vs. 21%, *p* = 0.05) (see Table 1). The only correlation between age and type of symptom was found for numbness: the presence of the symptom is associated with a greater age (medians 50 vs. 43, *p* = 0.03).

### Smell and Taste Dysfunctions

The characteristics of taste and smell dysfunctions have been analyzed more in depth (see Table 2): temporal onset in most of the sample is, on average, 6 days after the beginning of other symptoms (smell in 62% of the group and taste in 68%); only for a few of them these disorders emerged before other symptoms (in 13% of subjects, both dysfunctions appeared on an average of 5 days before other complaints). The type of dysfunction has also been explored, considering both sensory alteration, loss, or reduction. Anosmia and ageusia are the most frequent reported dysfunction particularly in case of smell (nearly 80% olfaction vs. 51% of taste, *p* = 0.004). No significant differences have been found in the rate of taste/smell resolution at the time of the interview (*p* = 0.18), though it resulted higher in case of taste problems (67% of taste vs. 52% of smell) and persistence of sensory disorders was similar for both senses (taste 5% and smell 7%).

**Table 2:** Analysis of the characteristics of smell and taste dysfunctions: temporal onset, type of dysfunctions, and resolution.

	**Smell dysfunction**	**Taste dysfunction**	***P*-value**
	**(*n* = 84)**	**(*n* = 79)**	
**Temporal onset**			
Before other COVID-19 symptoms	11 (13.4%)	10 (13.2%)	0.64
Together with other COVID-19 symptoms	20 (24.4%)	14 (18.4%)	
After the emergence of COVID-19	51 (62.2%)	52 (68.4%)	
Not available	2 (2.4%)	3 (3.8%)	
**Type of dysfunction**			
Sensory alteration	6 (7.2%)	17 (21.5%)	0.004
Loss	66 (79.5%)	40 (50.6%)	
Reduction	11 (13.3%)	22 (27.8%)	
Not available	1 (1.2%)	0	
**Resolution at the time of interview**			
Yes completely	44 (52.4%)	52 (66.7%)	0.18
Yes partially	34 (40.5%)	22 (28.2%)	
No	6 (7.1%)	4 (5.1%)	
Not available	0	1 (1.3%)	

At the time of the interview, the most frequent symptoms in the “COVID-19 negative with symptoms” group (*n* = 38), remained “smell dysfunction” (*n* = 21, 55%), and “taste dysfunction” (*n* = 12, 32%).

### DT at the Period of COVID-19 Positivity (COVID-DT)

As far as the emotional state reported by subjects, the DT showed a median score of 7 out of 10 when referring to the period of the COVID-19 illness. Gender and age were not associated with DT at the period of COVID-19 positivity (COVID-DT). Eighty-six percent of the sample declared moderate to high distress, and the most frequent manifestations of psychological symptoms were anxiety, irritability, negative mood, and a sense of loneliness (see Table 3). In addition, COVID-DT correlates significantly with the presence of some COVID-19 symptoms (fatigue, diarrhea, taste loss) and patients with more than seven symptoms (median value of symptom prevalence) showed higher levels of distress (see Table 3). While the loss of smell does not appear to be related to greater distress, in patients who have lost the taste, the level of distress is significantly higher in the period of illness. This result remained confirmed in the multivariate analysis (Beta = 1.58, 95% CI:0.15; 3.00, *p* = 0.02, see Supplementary Table 1).

**Table 3:** Distress thermometer scores at the time of COVID illness.

	**Total cohort (*n* = 104)**		
**DT scores**			
<5	24 (23.3%)		
≥5	79 (76.7%)		
Not available	2 (1.9%)		
Median DT score (min-max)	7 (0–10)		
	**DT score** **<5**	**DT score** **≥5**	***P*****-value**
	**(*****n*** **=** **25)**	**(*****n*** **=** **79)**	
**Demographics**			
Gender			
Male	8 (76.1%)	25 (75.8%)	0.97
Female	17(23.9%)	54 (24.2%)	
Age			
Median (min-max)	44 (24–61)	43 (23–65)	
**Emotional states**			0.86
Anxiety	5 (20.0%)	62 (78.5%)	<0.001
Nervousness	13 (52.0%)	61 (77.2%)	0.02
Irritability	6 (24.0%)	38 (48.1%)	0.03
Fear	7 (28.0%)	53 (68.0%)	<0.001
Sleep disorders	7 (28.0%)	43 (54.5%)	0.02
Negative mood	5 (20.0%)	52 (65.8%)	<0.001
Relationship	1 (4.0%)	20 (25.3%)	0.02
Pain	7 (28.0%)	38 (48.7%)	0.07
Nausea/constipation/diarrhea	5 (20.0%)	28 (35.4%)	0.15
Olfactory dysfunctions	13 (52.0%)	54 (68.4%)	0.14
Taste dysfunctions	10 (40.0%)	54 (68.4%)	0.01
Concentration	6 (24.0%)	39 (49.4%)	0.03
Loneliness	2 (8.0%)	41 (51.9%)	<0.001
**COVID-19 symptoms**			
Fatigue	16 (66.7%)	69 (84.5%)	0.01
Diarrhea	3 (12.5%)	25 (31.7%)	0.05
Loss of taste	14 (58.3%)	65 (82.3%)	0.02
Number of COVID symptoms ≥7	10 (41.7%)	52 (65.8%)	0.03

### HADS at the Time of Interview

Subjects' psychological state has been specifically investigated also through the HADS. Answers to the HADS questionnaire revealed the presence of anxiety and depression in a small percentage of cases at the time of the interview (score ≥11 <13% for anxiety and 6% for depression). Age and gender were not associate with HADS scores.

Looking at the relationship between the scores on the HADS scale and the loss of taste/smell, anxiety results to be significantly higher in patients with alterations in both senses than in subjects without these disorders. On the other hand, subjects who report loss of taste constantly refer also symptoms of depression, while in case of smell loss there is only a tendency, though not significant, to report more depressive symptoms (see Tables 4a,b,c). However, in multivariate analysis, the only factor associated at the limit of significance, with anxiety/depression disorders was sex: men tend to suffer less from these disorders than women (see Supplementary Tables 2, 3).

**TABLE 4a:** Hospital anxiety distress scores and olfactory/taste dysfunctions.

	**Total cohort (*n* = 104)**	**Male (*n* = 33)**	**Female (*n* = 71)**	***P*-value**
**HADS-A scores**				
<8	72 (69.9%)	27 (81.8%)	45 (64.3%)	0.10
8–10	18 (17.5%)	5 (15.2%)	13 (18.6%)	
≥11	13 (12.6%)	1 (3.0%)	12 (17.1%)	
Not available	1 (1.0%)	0 (0.0%)	1 (1.4%)	
Median HADS-A score (min-max)	5 (0–18)	3 (0–16)	6 (0–18)	
**HADS-D scores**				
<8	83 (80.6%)	30 (90.9%)	53 (75.7%)	0.19
8–11	13 (12.6%)	2 (6.1%)	11 (15.7%)	
≥11	7 (6.8%)	1 (3.3%)	6 (8.6%)	
Not available	1 (1.0%)	0 (0.0%)	1 (1.4%)	
Median HADS-D score (min-max)	3 (0–18)	3 (0–18)	4 (0–14)	
**HADS total scores**				
<13	73 (70.8%)	28 (84.8%)	45 (64.3%)	0.06
≥13	30 (29.1%)	5 (15.2%)	25 (35.7%)	
Not available	1 (1.0%)	0 (0.0%)	1 (1.4%)	
Median HADS total score (min-max)	8 (0–34)	5 (1–34)	9 (0–31)	

**TABLE 4b:** Hospital anxiety distress scores and olfactory/taste dysfunctions.

	**No olfactory dysfunction (*n* = 19)**	**Olfactory dysfunction (*n* = 84)**	***P*-value**
**Olfactory dysfunctions**			
**HADS-A score**			
Mean (SD)	3.7 (3.6)	6.0 (4.2)	0.018
Median (min-max)	2 (0–12)	5 (0–18)	
**HADS-D score**			
Mean (SD)	3.8 (3.1)	4.7 (3.6)	0.31
Median (Min-Max)	3 (1–13)	4 (0–18)	
**HADS total**			
Mean (SD)	7.5 (6.1)	10.6 (7.4)	0.06
Median (min-max)	5 (1–23)	8 (0–34)	
**COVID-DT**			
Mean (SD)	5.9 (2.8)	6.6 (2.5)	0.34
Median (min-max)	7 (2–10)	7 (0–10)	
**Current DT**			
Mean (SD)	3.4 (3.7)	4.1 (2.3)	0.29
Median (min-max)	2 (0–10)	4 (0–10)	

**TABLE 4c:** Hospital anxiety distress scores and olfactory/taste dysfunctions.

**Taste dysfunctions**	**No taste dysfunction** **(*n* = 25)**	**Taste dysfunction** **(*n* = 78)**	***P*-value**
**HADS-A**			
Mean (SD)	3.4 (2.8)	6.3 (4.3)	0.002
Median (min-max)	3 (0–12)	6 (0–18)	
**HADS-D**			
Mean (SD)	3.2 (2.5)	5.0 (3.8)	0.02
Median (min-max)	2 (1–9)	4 (0–18)	
**HADS total**			
Mean (SD)	6.6 (4.3)	11.2 (7.7)	0.009
Median (min-max)	6 (1–20)	9 (0–34)	
**COVID-DT**			
Mean (SD)	5.3 (2.4)	6.9 (2.8)	0.005
Median (min-max)	6 (2–9)	7 (0–10)	
**Current DT**			
Mean (SD)	2.8 (2.7)	4.4 (3.0)	0.03
Median (min-max)	2 (0–8)	5 (0–10)	

### Correlation Among Psychological Distress Scales

To complete the analyses of subjects' psychological state, results from the two administration of the DT (i.e., COVID-TD and Current-DT) have been compared, as well as the HADS total score with the Current-DT. As shown in Table 5, all psychological distress scales were correlated. In particular, at the Current-DT, subjects gained scores significantly lower than those obtained in the COVID period (median DT 4 and 7, respectively, *p* < 0.001) even though 53 subjects (51%) reached scores of clinical interest (DT ≥4: moderate and high distress). A strong association has also been found between the CurrentpDT and the HADS questionnaire: 86% of subjects who reached a high HADS total score (HADS >13) have also gained a critical Current-DT score (DT >5), while only 29% of subjects with lower HADS scores (HADS <13) have an high DT score (*p* < 0.001) (see Supplementary Table 4). Accordingly, results from the administration of HADS confirm that 30 out of the all cohort (29%) would require clinical psychological intervention as shown by their overall HADS score (≥13).

**TABLE 5:** Correlations between different psycogical distress scores.

**Comparison**	**Spearman linear coefficient**	***P*-value**
COVID-DT vs. Current COVID	rho = 0.60	<0.001
COVID-DT vs. HADS-A	rho = 0.55	<0.001
COVID-DT vs. HADS-D	rho = 0.49	<0.001
COVID-DT vs. HADS total	rho = 0.56	<0.001

## Discussion

This study evidences that smell and taste dysfunctions in subjects with mild to moderate COVID-19 symptoms are associated with higher levels of psychological distress compared to those not experiencing taste and smell dysfunctions [as observed by (25, 36)]. Anxiety and depressive symptoms are the most frequent responses, even though depression appears to be higher in relation to ageusia with respect to anosmia. Indeed, it is well-known that olfactory and taste sensory loss can have a negative impact on emotional well-being and quality of life, leading to feelings of loneliness, fear, and depression, as well as difficulties concerning social/sexual relationships (25, 36). Since taste and smell have always been a fundamental need in evolution and survival, psychological effects can be easily expected (25, 36–38). Consistent with other findings [e.g., (39)], women were more frequently affected by smell and taste disorders compared with men.

The COVID-19 pandemic issues such as the high risk of infection and reinfection, inadequate protection from contamination, overwork, frustration (i.e., witnessing death and feeling powerless over the levels of patient death, the delivery of patient care), discrimination, isolation, patients with negative emotions, lack of contact with families, exhaustion, the incapacity of healthcare systems to respond to the increased demand (40–43) play an important role in patients' emotional experience, the co-existence of COVID-19 symptoms however may intensify their reactions and for the most induce anxiety disorders. In addition, even when people are healed, they continue to experience high emotional distress, especially because they are worried of getting sick again. In accordance with Lima et al. (44) and Duan and Zhu (45), this suggests the need for appropriate psychological interventions under pandemic conditions to prevent people from developing more serious or long-lasting psychological disorders and, as far as HCW, to reduce the risk of work related distress (42, 46–49).

## Limitations

The study has some limitations. Data analysis was retrospective and did not fulfill the criteria of a randomized controlled study. Indeed, it is possible that individuals who adhered to be interviewed and completed the questionnaires were those who experienced greater psychological distress during the pandemic or who are simply more interested in psychological factors and implications. Another limitation might pertain the validity of the selected tools to investigate emotional distress related to the COVID pandemics. The DT and the HADS have been chosen as they are among the most widely used tests for screening and evaluating distress associated with organic diseases; however, they are not specifically designed for assessing emotional reactions during epidemics and/or pandemics, or for detecting their evolution over time as the pandemic continues; in addition, responses of COVID-DT can be influenced by recall bias. Finally, individuals' stress intensity has not been evaluated considering the duration of taste and smell disorders but taking into account only their presence or absence at the time of the interview. This aspect certainly deserves attention and could be investigated in-depth in future studies.

## Conclusion

Anosmia and dysgeusia in people infected with COVID-19 are confirmed to increase psychological distress. Smell and taste dysfunctions can amplify psychological distress, and depressive symptoms are mostly associated with the loss of taste and tend to persist even after resolution of symptoms and disease. In this study, HCW reported high rates of emotional distress associated with COVID-19 infection and taste and smell dysfunctions. This highlights the importance of developing a systematic approach to identify at-risk individuals to prevent the development of more serious or long-lasting psychological disorders. Psychological interventions targeting HCW who contracted the COVID-19 infection, should be designed considering the symptom effects and job reintegration, which should take into account anxiety and depression experienced by workers who became ill. Interventions to benefit HCW, such as those suggested in the literature of psychological support, CBT, self-help strategies, and others, could be targeted to COVID-19 psychological effects. As well as the importance of collaboration in health care provider support services between occupational doctors, occupational psychologists, and clinicians to study work-related risks.

## Data Availability Statement

The raw data supporting the conclusions of this article will be made available by the authors, without undue reservation.

## Ethics Statement

The studies involving human participants were reviewed and approved by Comitato Etico Unico Regionale ARCS- FVG Nr. 0000611-P del 23/06/2020. The patients/participants provided their written informed consent to participate in this study.

## Author Contributions

LD was the main author and lead researcher. CC and AL assisted in the conception of the research, recruitment of subjects, collection of data, and contributed to the writing-up of the manuscript. FG carried out statistical analyses of results and contributed to interpretation of the final conclusions. GA, IS, VB, and MP helped in the recruitment of subjects and carried out the interviews. VP, PM, FR, BG, and CN supervised the project and discussed the findings of this work. All authors contributed to the article and approved the submitted version.

## Conflict of Interest

The authors declare that the research was conducted in the absence of any commercial or financial relationships that could be construed as a potential conflict of interest.
