# Supplementary material for: Investigation on the Loss of Taste and Smell and Consequent Psychological Effects: A Cross-Sectional Study on Healthcare Workers Who Contracted the COVID-19 Infection
Source: Front Public Health. 2021 May 28;9:666442. doi: 10.3389/fpubh.2021.666442 (PMC8193118; doi:10.3389/fpubh.2021.666442)
Supplement: Supplementary file 1 [file Data_Sheet_1.docx]

Supplementary Material: tables

**Supplementary Table 1** Risk factors for Distress according to DT scores at period’s illness

| **Variables** | **Beta (95% CI)** | **p value** |
| --- | --- | --- |
| Age | 0.02 (-0.03;0.06) | 0.39 |
| Gender (Male vs Female) | -0.10 (-1.18;0.97) | 0.84 |
| Number of Symptoms (≥7 vs <7) | 0.55 (-0.53;1.64) | 0.31 |
| **Loss taste (yes vs no)** | **1.58 (0.15;3.00)** | **0.02** |
| Loss smell  (yes vs no) | -0.34 (-1.84;1.19) | 0.66 |

**Supplementary Table 2** Risk factors for anxiety according to HADS-A score at the time of Interview

| **Variables** | **Beta (95% CI)** | **p value** |
| --- | --- | --- |
| Age | -0.01 (-0.09;0.06) | 0.72 |
| Gender (Male vs Female) | -1.64 (-3.39;0.10) | 0.07 |
| Number of Symptoms (≥ 7 vs <7) | 1.19 (-0.57;2.96) | 0.18 |
| Loss taste (yes vs no) | 1.74 (-0.57;4.07) | 0.13 |
| Loss smell  (yes vs no) | 0.24 (-2.23;2.73) | 0.84 |

**Supplementary Table 3** Risk factors for Depression according to HADS-D score at the time of Interview

| **Variables** | **Beta (95% CI)** | **p value** |
| --- | --- | --- |
| Age | 0.01 (-0.05;0.07) | 0.77 |
| **Gender (Male vs Female)** | **-1.47 (-3.00;0.05)** | **0.05** |
| Number of Symptoms (≥ 7 vs <7) | 0.21 (-1.34;1.75) | 0.79 |
| Loss taste (yes vs no) | 1.54 (-0.49;3.57) | 0.14 |
| Loss smell  (yes vs no) | -0.50 (-2.68;1.68) | 0.65 |

**Supplementary Table 4.** Association between Distress and HADS-Total Scores at time of Interview

| **Current DT**  **Scores**  **<5**  **≥ 5** | **HADS Total**    **Scores**  **<13 ≥13**  52 (71.2%) 4 (13.8%)    21 (28.8%) 25 (86.2%) | **p value**  **p<0.001** |
| --- | --- | --- |

*for 2 patients no data regarding Current DT
